# Egg donor self-reports of ovarian hyperstimulation syndrome: severity by trigger type, oocytes retrieved, and prior history

**DOI:** 10.1007/s10815-023-02855-3

**Published:** 2023-06-22

**Authors:** Diane M. Tober, Kevin Richter, Dougie Zubizarreta, Said Daneshmand

**Affiliations:** 1 Department of Anthropology/Institute for Social Science Research, University of Alabama, 24b Ten Hoor Hall, Tuscaloosa, AL 35487 USA; 2 Institute for Health and Aging, University of California, San Francisco, 490 Illinois St, Box 0646, San Francisco, CA 94158 USA; 3 The Fertility Scientist, Silver Spring, MD 20910 USA; 4 School of Public Health, Harvard University, Boston, MA 02115 USA; 5 San Diego Fertility Center, San Diego, CA 92130 USA

**Keywords:** Egg donation, Ovarian hyperstimulation syndrome (OHSS), Ovarian stimulation

## Abstract

**Purpose:**

To evaluate self-reported survey data provided by US oocyte donors on their experiences with ovarian hyperstimulation syndrome and possible correlations between OHSS severity and number of oocytes retrieved, trigger type, and prior OHSS history.

**Methods:**

An 85-question retrospective survey was administered online. Survey questions included demographic information, reasons for donating, immediate per-cycle experiences and outcomes, perceptions of informed consent, and perceived impact of donation on long-term health. Quantitative Data for this study was collected between February 2019 and September 2020 via Qualtrics^XM^ (January 2019), an online survey platform. Follow-up interviews were also conducted. Participants were recruited via fertility clinics, egg donation agencies, and online forum. The research was approved by the University of California, San Francisco Institutional Review Board (#14-14765).

**Results:**

Of 420 initiated US oocyte donor online surveys, 289 (68%) respondents provided detailed information on per cycle experiences with ovarian hyperstimulation syndrome, number of oocytes retrieved, and trigger type over a total of 801 cycles. On cycles where donors reported receiving GnRH agonist triggers (*n* = 337), they reported milder OHSS compared to cycles with hCG or dual triggers. Among donors undergoing multiple retrieval cycles, the severity of OHSS in second cycles was strongly associated with OHSS severity in first cycles.

**Conclusion:**

Self-reported OHSS in oocyte donors is lower in GnRH antagonist stimulation protocols combined with GnRHa trigger and in cycles where donors reported fewer than 30 oocytes retrieved. Donors who reported severe OHSS on a prior cycle were significantly more likely to experience severe OHSS on a subsequent cycle.

## Introduction

In the USA, a reported 49,193 donor oocyte retrievals were performed between 2016 and 2017, including 17,099 unique oocyte donors [1]. Fresh donor oocytes are reportedly used in roughly 12% of all ART cycles performed in the USA [2]. Despite the wide use of donor oocytes in in ART cycles, there is still little information on immediate health outcomes for oocyte donors compared to others undergoing controlled ovarian stimulation (COS) for their own fertility treatment. With the growing demand for donor oocytes, as well as changing egg donation practices due to oocyte vitrification, enhancing knowledge on donor experiences and outcomes is crucial.

The most common immediate complication for oocyte donors is ovarian hyperstimulation syndrome (OHSS). OHSS is a potentially life-threatening iatrogenic complication associated with controlled ovarian stimulation [3]. Individuals may undergo COS to produce oocytes for use in their own in vitro fertilization (IVF) treatment, to preserve oocytes for their own future use, or as an oocyte donor to help someone else conceive a child. The condition can be classified as mild, moderate, severe, and in rare cases can become critical. Symptoms range from mild bloating to, in its most severe forms, rapid weight gain, extreme abdominal bloating requiring paracentesis, nausea and vomiting, and syncope. Critical OHSS can result in respiratory distress and renal failure [4–8].

Studies estimate that severe OHSS occurs in roughly 1–10% of all ovarian stimulation cycles and may vary according to medical protocols used for ovarian stimulation and triggering [4, 9–11]. Some studies have found a higher risk for critical OHSS among hospitalized IVF patients with other comorbidities, such as advanced maternal age, non-Hispanic Black ancestry, and PCOS [8]. Others have found risk for moderate to severe OHSS to be higher for people who are younger, have lower body mass index (BMI), high resting antral follicle counts, polycystic ovaries, and may also vary according to ancestry [10, 12–15]. Young women with lower BMI and healthy antral follicle counts are the primary egg donor population, yet studies that specifically focus on OHSS in egg donors is scant, especially when compared to fertility patients who undergo COS to achieve pregnancy.

In one of the few studies specifically focusing on OHSS in oocyte donors, researchers found a 1.5% risk of severe OHSS and a 33.5% risk of moderate OHSS among 149 donors over 400 egg retrieval cycles [16]. Another clinical study of 587 oocyte donors at a single IVF center found 9% of cycles had to be cancelled due to OHSS, out of caution for donor health [17]. Another retrospective survey study of 246 US compensated oocyte donors examined donor experiences with pain and noted that 13.4% of donors in their study reported OHSS, among other complications, but the severity of OHSS is not discussed [18]. One study on perceptions of informed consent among oocyte donors found that 30% felt uninformed about OHSS and over 66% felt that their post-donation physical experiences did not match their expectations based upon what they were told during the informed consent process [19]. Identifying risk factors for OHSS among oocyte donors, and avenues for mitigating risk, is especially important for this population, who undergo an elective medical procedure with no medical benefit.

Over the past 25 years, medical protocols have been developed that reduce the occurrence of OHSS in patients undergoing COS, such as administering a GnRH agonist trigger rather than a trigger containing hCG. However, OHSS hospitalization rates have still not significantly declined as would be expected if safer protocols were widely adopted [20]. It is thus vital to gain a better understanding of the occurrence and severity of OHSS among oocyte donors, potential risk factors for OHSS that may be unique to the donor population, or other factors that may influence OHSS occurrence such as stimulation protocol preference among practitioners. Examining possible linkages between stimulation protocols, donor oocyte production, and frequency and severity of OHSS can help inform clinical practices to reduce or eliminate its occurrence in the donor population. Donor self-reports of experiences with OHSS are important to consider as severity varies, and often, cases may not be reported to or managed by the clinic where donation occurred. To the best of our knowledge, this study is the first to examine the degree to which oocyte donors experience OHSS and possible associations between OHSS severity and trigger type, oocyte quantity, and prior OHSS history.

## Materials and methods

An 85-question survey was administered online. Survey questions included demographic information, reasons for donating, immediate per-cycle experiences and outcomes, perceptions of informed consent, and perceived impact of donation on long-term health. Data for this study was collected between February 2019 and September 2020 via Qualtrics^XM^ (January 2019), an online survey platform. Survey participants were offered the opportunity to participate in follow-up, semi-structured, open-ended interviews. Of the 289 survey respondents during the study period, 202 indicated that they would like to be interviewed. Within this timeframe, we were able to conduct semi-structured, open-ended interviews with 96 participants. Each of these interviews lasted between 1 and 3 h. For the purposes of this paper, the interviews serve to confirm survey accuracy as well as shed further light on the experiences of those who reported experiencing “Critical OHSS.” The research was approved by the University of California, San Francisco Institutional Review Board (#14-14765).

Research participants were recruited via online forum, social media, infertility clinics, egg donation agencies, and word of mouth. Announcements circulated about the study provided a link to the research website (eggdonorresearch.org) where prospective participants could find additional information about the study, click on the survey link, and answer preliminary screening questions to receive the survey password from the research team. A total of 532 oocyte donors initiated the survey. The survey took between 20 min and an hour and varied according to the number of cycles a donor completed. Respondents who resided or donated outside the USA (*n* = 88) were excluded from this analysis as were voluntary unpaid donors (*n* = 46). Of the remaining 398, 289 (72%) answered survey questions pertaining to per-cycle outcomes.

**Fig. 1:**
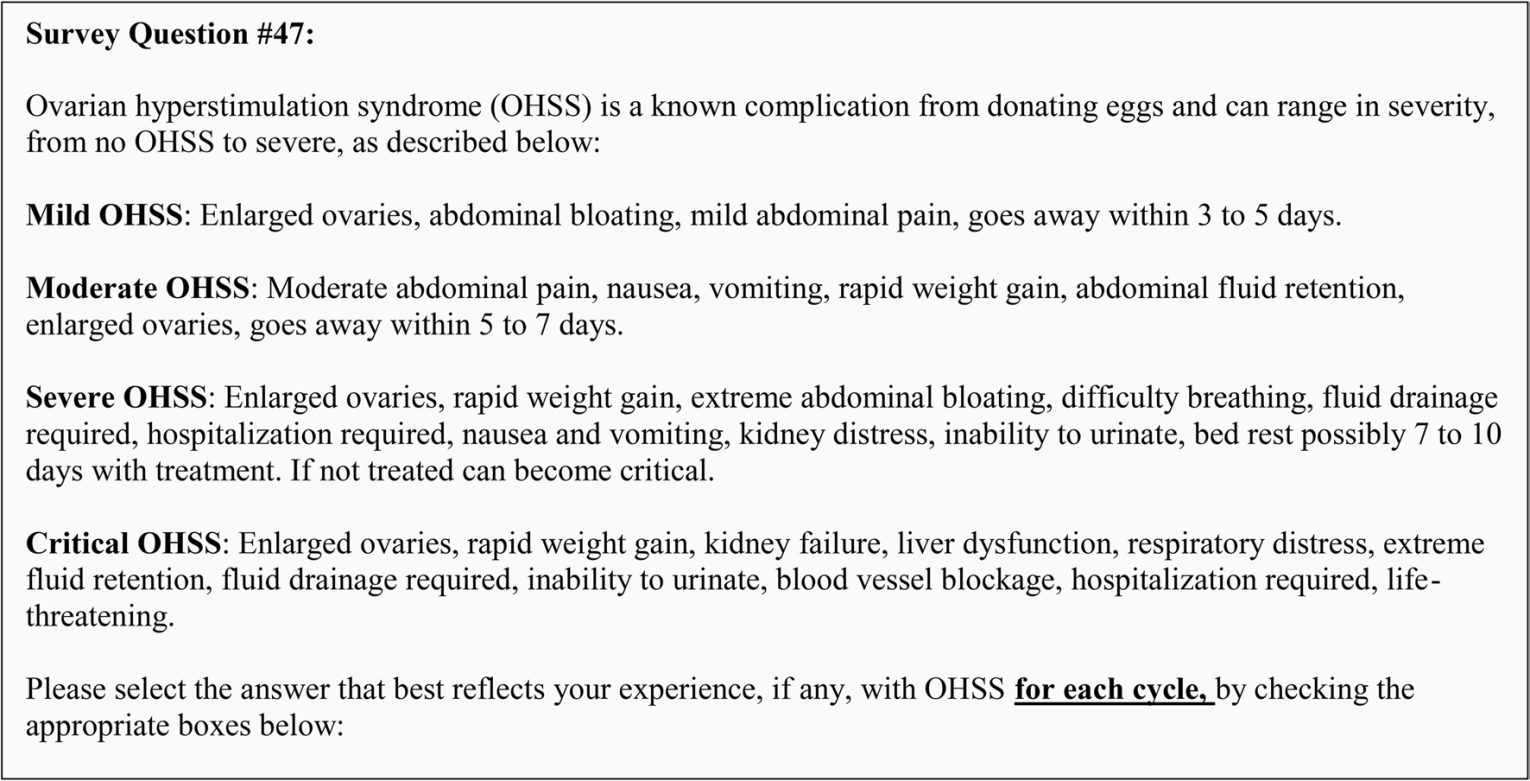
OHSS survey question and OHSS classifications

The survey collected self-reported data on numbers of eggs retrieved per cycle, trigger type (hCG, GnRHa/Lupron™, or combined (dual)), and severity of OHSS. The survey defined ovarian hyperstimulation syndrome according to ASRM classifications for mild, moderate, severe, and critical, but in lay language, as seen as follows (Fig. 1) (2). Respondents were instructed to identify their experience, or not, with OHSS by checking the box which most closely fit their experience for each stimulation cycle, from “No OHSS to Critical OHSS.” Text boxes were included so respondents could provide additional information. The survey question, including OHSS classifications can be found as follows.

Participants were able to further refine their responses by checking appropriate boxes on per-cycle post-donation symptoms checklists that included mild, moderate, and severe “bloating,” “bed rest,” “hospitalization,” “difficulty breathing,” “nausea and/or vomiting,” “drained fluids” (paracentesis), abdominal pain, pain in ovary, and other symptoms associated with OHSS. This allowed us to cross-check perceived experience with OHSS and OHSS symptom severity to enhance data accuracy. Text boxes were also provided so survey respondents could include additional information about their experiences.

Descriptive quantitative statistics of central tendency and dispersion are reported both parametrically (mean and standard deviation (SD)) and non-parametrically (median, range, interquartile range (IQR), and interdecile range (IDR)). The severity of OHSS was converted to a five-level ordinal scale (none = 0, mild = 1, moderate = 2, severe = 3, critical = 4) for statistical analysis. Statistical comparison of OHSS severity among all three trigger type groups was performed nonparametrically using Kruskal-Wallis analysis of variance (ANOVA) on ranks, followed by Mann-Whitney *U* tests for pairwise comparisons. Parametric analysis of variance (ANOVA) was used to compare numbers of oocytes retrieved among the three trigger-type groups, followed by post hoc Tukey’s honestly significant difference (HSD) tests for pairwise comparisons of oocyte yields.

## Results

### Quantitative and categorical demographic characteristics

A total of 289 compensated current and former US egg donors responded to per cycle survey questions on number of donation cycles completed, number of eggs retrieved, trigger medication used, and symptoms and experiences with OHSS. Respondents reported undergoing a total of 801 donation cycles, with a range from one to 13 cycles, and an average of 3.0 cycles per donor. Mean number of oocytes per cycle was 26.5, with a range of 0 (e.g., for cancelled cycles) to a high of 80 on a single cycle.

Participants ranged in age from 18 to 57 at the time of survey completion, with an average age of 25.1 at first donation. Several older respondents donated eggs 11 years or more prior to taking the survey, but these were in the minority (*n* = 44, or 15.21%). The majority (45.67%) had donated within twelve months prior to taking the survey. Information on clinic type (e.g., academic, private, egg banks) was also recorded to see if we could identify any trends by clinic type. However, most repeat donors—especially those who went through egg donation agencies—donated through different clinics or types of practices throughout their donation careers, so data on donation outcomes by clinic type is inconclusive. Most (71%) identified as White only, and 75% were in college or achieved a bachelor’s degree or higher (Table 1). While not a very diverse sample, donor reported ancestry is, for the most part, reflective of the population of women who are recruited to become egg donors in the USA.

**Table 1:** Quantitative and categorical demographic characteristics

Demographics	Mean	SD	Median	IQR	IDR	Min	Max
Age at first donation	25.1	3.4	25	23–27	21–30	18	36
Age at last donation	26.7	3.4	27	24–29	23–31	18	36
Number of donation cycles	3.0	2.3	2	1–4	1–6	1	13
Years since last donation	4.8	6.6	2	1–6	0–17	0	27
Oocytes per cycle	26.5	12.7	25	18–33	13–42	0	80
Time since last donation (*n* = 289)							
0–11 months	132			45.67%			
1–3 years	62			21.45%			
4–6 years	31			10.72%			
7–10 years	20			6.92%			
11–15 years	11			3.80%			
> 15 years	33			11.41%			
				Number*	Percentage*		
Race/ancestry	European/White			265 (205)	92% (71%)		
	East Asian (e.g., Chinese, Japanese, Korean)			18 (10)	6% (3%)		
	Hispanic/Latinx			17 (5)	6% (2%)		
	Jewish			17 (0)	6%		
	African American/Black, Afro-Caribbean			14 (2)	5% (0.7%)		
	Native American or Alaskan			13 (0)	4%		
	Southeast Asian (e.g., Thai, Filipina, Indonesian)			8 (2)	3% (0.7%)		
	Pacific Islander, native Hawaiian, Polynesian			4 (1)	1.4% (0.3%)		
	South Asian (e.g., India, Pakistan, Nepal, Sri Lanka)			2 (1)	0.7% (0.3%)		
	West Asian (e.g., Iranian, Afghan, Arab, Turkish)			2 (0)	0.7%		
	Other			6 (1)	2% (0.3%)		
Education	High school/GED			39	13%		
	Vocational/technical			34	12%		
	Bachelor’s degree			139	48%		
	Graduate degree			77	27%		

*Values in parentheses indicate respondents listing a single racial/ethnic category.

### OHSS incidence per oocyte donor

Of the 289 unique oocyte donors included in our analysis, 13% reported no OHSS symptoms in any of their donation cycles, and 35% experienced only mild symptoms considered within the range of a normal response to controlled ovarian stimulation (COS). Moderate OHSS was the most common, with 39% of donors reporting this condition. Thirty-four donors (12%) reported severe OHSS in at least one donation cycle, and five (1.38%) experienced critical OHSS.

### OHSS incidence per oocyte donation cycle

**Fig. 2:**
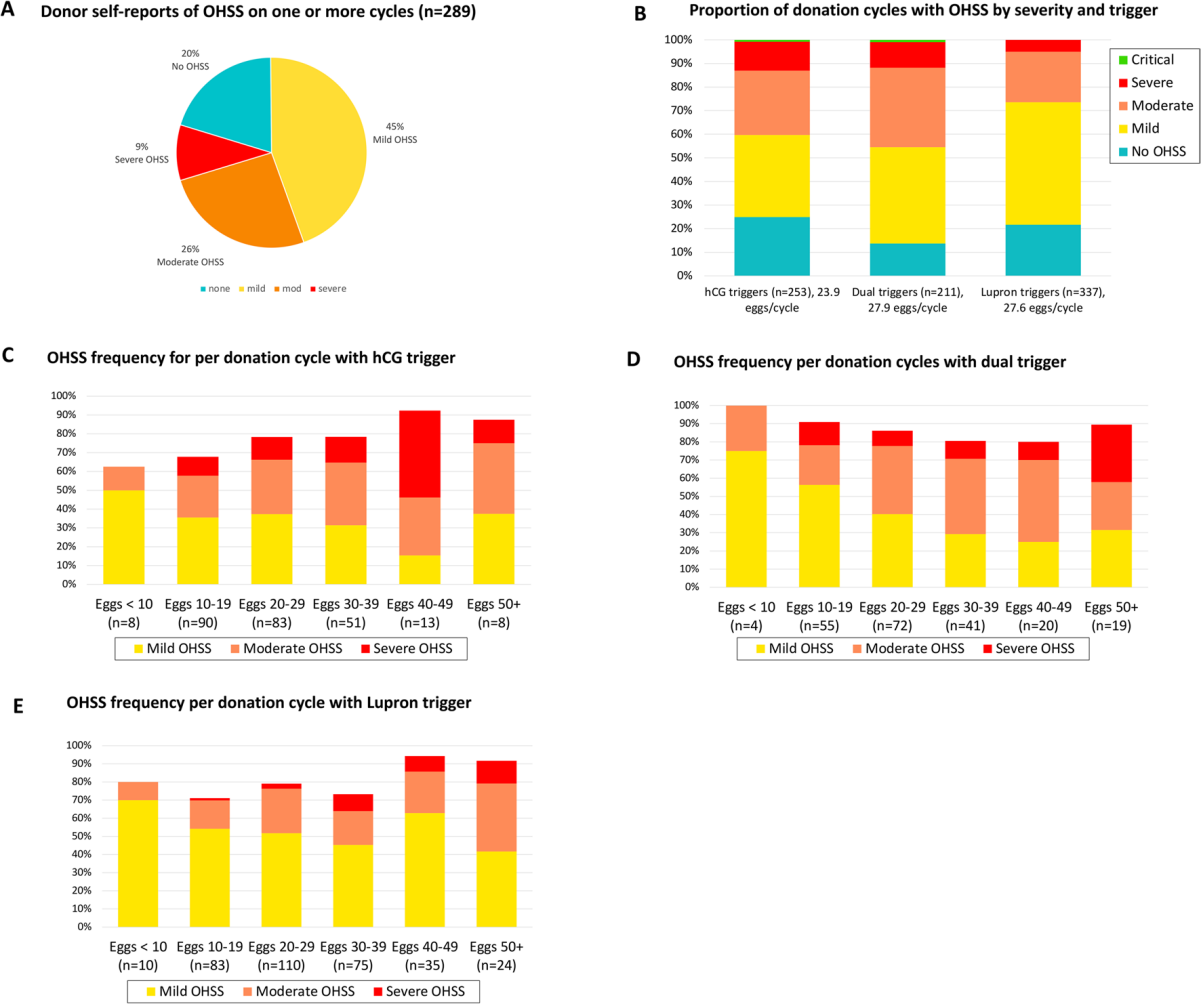
OHSS incidence per oocyte donation cycle

When calculated on a per cycle bases, out of 801 total reported cycles (Fig. 2A), most resulted in only mild OHSS symptoms (45%) or no OHSS symptoms at all (20%). Moderate OHSS was reported for 26% of donation cycles. Severe OHSS occurred in 9% of cycles and another four (0.5%) resulted in critical OHSS. Donors reported 253 cycles triggered with hCG alone, 337 with a GnRHa (usually Lupron™) alone, and 211 with hCG and GnRHa combined (i.e., dual triggers). GnRHa triggers were associated with significantly reduced OHSS symptoms compared to hCG or dual triggers (Fig. 2B, *p* = 0.0002 for 3-group comparison, *p* = 0.03 for GnRHa vs hCG, *p* < 0.0001 for GnRHa vs dual). Approximately 14% fewer oocytes were retrieved with hCG trigger vs either GnRHa or dual triggers (*p* = 0.0004 for 3-group comparison, mean = 23.9 for hCG vs mean = 27.6 for GnRHa, *p* = 0.0014, and mean = 27.9 for dual triggers, *p* = 0.0024).

### OHSS incidence per donation cycle, by trigger type

Adjusting for the smaller oocyte cohort sizes associated with hCG trigger, severe OHSS was most common after hCG trigger (Fig. 2C, 10–12% with retrieval of 10 to 39 oocytes, and 19% with retrieval of 40 oocytes or more). Severe OHSS was reported to occur in 5–7% of cycles with retrieval of 10 to 49 oocytes following dual trigger, and 26% when 50 or more oocytes retrieved (Fig. 2D). OHSS was much milder with GnRHa trigger, with severe OHSS occurring only about 1% of the time when fewer than 30 oocytes were retrieved (Fig. 2E). However, reported severe OHSS increased to 4% with retrieval of 30 to 39 oocytes, and 6.8% of cycles with 40 or more oocytes—even in the donors who reported a Lupron-only trigger. There were no accounts of severe OHSS among any cycles with retrieval of fewer than 10 oocytes, regardless of trigger type.

### OHSS correlation between 1st and 2nd donations

**Fig. 3:**
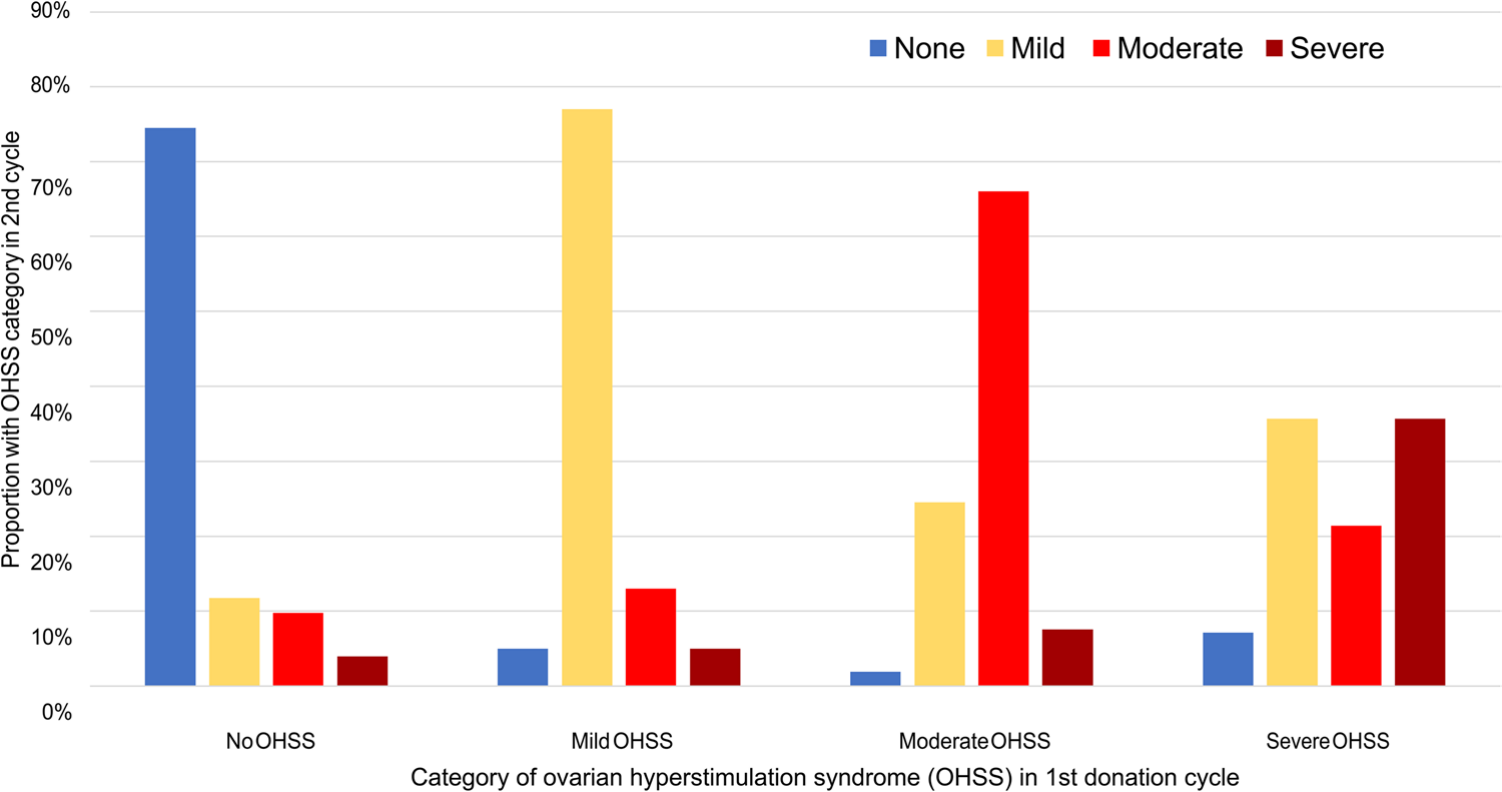
OHSS severity in first versus second donation cycles

Three-quarters of all surveyed donors (218 of 289) underwent at least one additional donation cycle after their first. We evaluated the severity of OHSS experienced in second and subsequent donations according to the degree of OHSS that these donors experienced in their first donation cycles. The degree of OHSS experienced in a first donation cycle was highly predictive of OHSS severity in subsequent donation cycles, with 155 donors (71%) reporting the same degree of OHSS in their second cycles as their first. Two-thirds of all donors reporting moderate OHSS symptoms in their first cycles also reported moderate OHSS in their second cycles, and three-quarters of all donors reporting either no OHSS or only mild OHSS in their first cycles reported the same degree of OHSS in their second cycles (Fig. 3). Fourteen of the 24 donors (58%) with severe OHSS in their first cycles donated at least once more. In their second cycles, five of these donors (36%) experienced severe OHSS again, and more than half (57%) had at least moderate OHSS symptoms. Non-parametric correlation analysis confirmed a strong and statistically significant association between OHSS severity in first versus second donation cycles (Spearman’s rank correlation, *r*_*s*_ = 0.608, *p* < 0.0001).

### Case studies of repeat donors who experienced severe OHSS

Subjects who donated multiple times and experienced severe OHSS in one or more of their donation cycles are listed in Table 2, along with number of retrieved oocytes, reported trigger type, and OHSS grade for each of their donation cycles.

**Table 2:** Donors who underwent multiple cycles and experienced severe OHSS one or more cycles, with numbers of oocytes retrieved, trigger type (*L* Lupron, *H* hCG, *D* Dual), and OHSS severity in all sequential donation cycles. “?” indicates data not reported

Donor	Age at 1st donation	Number of donations	Oocytes	Trigger Type	OHSS
2	24	5	22, 21, 34, 19, 28	H, H, H, H, H	Moderate, severe, moderate (× 3)
16	23	6	36, 26, 23, 25, 28, 24	L, L, L, L, L, L	Severe, mild (× 5)
27	23	6	35, 35, 35, 35, 35, 35	L, L, H, H, L, L	Severe, moderate, mild (× 4)
29	23	5	16, 19, 22, 16, 17	L, H, H, D, D	Mild, severe, moderate, mild (× 2)
35	23	6	26, 32, 28, 41, 36, 30	L, L, L, L, L, L	Moderate (× 3), severe, moderate (× 2)
41	25	3	34, 47, 44	L, L, D	Mild, mild, severe
50	23	5	15, 20, 26, 33, 40	H, H, H, D, H	Moderate (× 3), severe, moderate
51	28	6	30, 12, 12, 26, 17, 6	H, D, D, D, D, D	Severe, mild (× 2), moderate, mild (× 2)
53	22	4	32, 32, 52, 32	D, D, D, D	Mild, severe, moderate (× 2)
61	21	2	11, 69	D, D	Moderate, severe
**64**	21	13	35, 35, 35, 30, 20, 36, 22, 19, 30, 30, 20, 25, **20**	H, H, H, H, H, H, H, H, L, L, D, ?, ?	None (× 10), moderate, mild, critical
65	27	2	15, 20	D, D	Mild, severe
83	27	2	?, ?	L, L	Mild, severe
89	19	6	28, 23, 58, 40, 15, 27	D, D, D, D, H, D	Mild (× 2), severe, mild (× 3)
99	20	10	15, 15, 18, 18, 18, 18, 18, 18, 18, 18	?, ?, ?, ?, ?, H, H, H, H, H	Moderate (× 5), severe (× 3), moderate (× 2)
140	21	5	26, 23, 13, 28, 39	D, D, D, D, D	Severe, mild, moderate (× 3)
**142**	23	3	26, 24, **22**	H, H, H	Severe, moderate, critical
164	24	2	?, 42	?, H	Severe, severe
175	27	3	38, 36, 30	L, H, H	None, severe, moderate
183	23	2	12, 18	?, ?	Moderate, severe
184	21	6	33, 45, 38, 30, 22, 40	D, L, D, D, D, L	Severe, mild, moderate (× 4)
201	26	2	20, 25	D, D	Mild, severe
206	21	2	11, 69	H, H	Moderate, severe
229	22	11	47, 38, 44, 53, 80, 63, 42, 37, 44, 40, 52	H, H, D, D, L, L, L, L, L, L, L	Severe (× 2), mild (× 9)
235	36	3	26, 18, 23	?, ?, ?	Severe, moderate, mild
238	21	6	24, 36, 45, 50, 50, 26	D, D, D, D, D, D	Mild (× 5), severe
242	25	2	41, 50	L, L	Severe, severe
247	32	3	36, 39, 19	H, H, H	Mild, moderate, severe
256	28	2	75, 52	D, D	Severe, severe
355	19	2	20, 18	D, D	Severe, severe
360	24	2	33, 45	L, L	None, severe
400	27	2	17, 14	L, L	Severe, none
**404**	26	8	12, 14, 12, 16, 12, 14, 16, 14	D, D, D, D, D, D, D, D	Mild (× 1), severe cycle 2, moderate (5–7), critical cycle 8
490	19	6	40, 25, 25, 25, 25, 25	?, ?, ?, ?, H, H	Severe, mild (× 5)

Donors who appear in bold text reported critical OHSS on at least one cycle

Six of the 14 donors who donated at least once more after experiencing severe OHSS in their first cycles had only mild or no OHSS symptoms in their second cycles, some of which appeared to be the possible result of treatment adjustments. One (donor 16) had severe OHSS in her first cycle after retrieval of 36 oocytes, but only mild OHSS in five subsequent cycles with retrieval of 23–28 oocytes (all Lupron triggers). Another (donor 51) had severe OHSS after her first hCG triggered retrieval of 30 oocytes, but only mild OHSS in four subsequent dual triggered cycles with retrieval of 6–17 oocytes (and one cycle with moderate OHSS after dual trigger and retrieval of 26 oocytes). A third (donor 184) had only mild OHSS with Lupron trigger (and retrieval of 45 oocytes) after a first cycle with dual trigger and retrieval of 33 oocytes that resulted in severe OHSS and moderate OHSS in subsequent dual triggered cycles with retrieval of fewer oocytes. A fourth (donor 490) experienced severe OHSS in her first cycle with retrieval of 40 oocytes, but only mild OHSS in five subsequent retrievals of 25 oocytes each. Donor 229 had severe OHSS in her first two cycles (both hCG triggered), but only mild OHSS in nine subsequent cycles with dual (2) or Lupron (7) triggers, despite consistently producing among the largest oocyte cohorts of any donor (mean = 50 per cycle, maximum = 80).

In most cases, changing from hCG to a Lupron or other GnRHa trigger, and lower egg yield, reduced incidence of severe OHSS in subsequent cycles. However, changes in trigger type or oocyte yield could not explain the mild OHSS observed in later cycles for some other donors (27, 140, and 400) after they experienced severe OHSS in their first donation cycles. Some outcomes were contrary to expectations. For example, donor 27 reported only mild OHSS in two hCG triggered cycles after having severe OHSS in an initial Lupron triggered cycle, with comparable oocyte yields. This could be potentially due to errors in recall.

Twenty donors experienced severe OHSS in one or more later donation cycles after having milder OHSS symptoms with their first donations. Eleven of these reported severe OHSS in their second cycles after no (2), mild (5), or moderate (4) OHSS in their first cycles. In three (donors 61, 206, and 360), many more oocytes were retrieved in the second cycles compared to the first (with no changes in trigger type). Two others (donors 29 and 175) experienced only mild or no OHSS symptoms in their first cycles (triggered with Lupron), but severe OHSS in their second (hCG triggered) donations despite having similar-sized oocyte cohorts. Both donors underwent third donations, both hCG triggered, and both with moderate OHSS, and one (donor 29) experienced only mild OHSS in two subsequent cycles (her fourth and fifth) when dual triggered. For the remaining six (donors 2, 53, 65, 83, 183, and 201), there were no apparent changes in trigger type or oocyte yield that could explain the severe OHSS in second cycles after milder OHSS in first cycles.

The other nine donors first presented with severe OHSS in their third (3), fourth (2), fifth (1), sixth (2), or later (1) cycles. A few of these cases may be explained by variation in trigger type or oocyte numbers. One (donor 41) had severe OHSS in her third (dual triggered) cycle after two Lupron triggered cycles with only mild OHSS. A second (donor 89) underwent five dual-triggered cycles and experienced severe OHSS in the one cycle (her third) with retrieval of more than twice as many oocytes as other cycles with only mild OHSS. A third (donor 35) underwent six Lupron-triggered cycles, all with moderate OHSS except for severe OHSS in the one cycle with retrieval of the most oocytes (41 vs 26-36).

Neither oocyte numbers nor trigger type could explain why the remaining six donors experienced their first episode of severe OHSS with their third donation or later, but not in earlier donations. In several cases, severe OHSS occurred in later donation cycles in the absence of, or despite, changes in trigger type or oocyte cohort size. One participant (donor 247) experienced severe OHSS in her third donation cycle with retrieval of only 19 oocytes, after first mild and then moderate OHSS with retrieval of 36 and 39 oocytes in her first and second donations, respectively. Donor 238 had severe OHSS in her sixth donation cycle with retrieval of 26 oocytes, after only mild OHSS in five earlier donations of 24 to 50 oocytes each. Another (donor 404), who consistently produced relatively small cohorts of 12 to 16 oocytes across eight donation cycles, experienced only mild OHSS in her first three donations, followed by moderate, severe, moderate, critical, and severe OHSS in the next five donations. Donor 64 underwent ten consecutive donation cycles, most with retrieval of 30 or more oocytes, with no symptoms of OHSS. However, the following three retrievals of 20, 25, and 20 oocytes were associated with moderate, mild, and critical OHSS.

### Four cases of critical OHSS

A total of four donors reported having been hospitalized with critical OHSS. One donor, MC, experienced this on her first cycle and elected not to undergo another donation, so she is not listed in Table 2. On the survey, MC reported hCG trigger, 20 eggs retrieved, and having been hospitalized due to OHSS. In a follow-up interview, she reported having experienced extreme pelvic pain several hours following her egg retrieval and fainting on the way to the bathroom. In her words, the experience felt like “My whole stomach and abdomen were on fire and pain was like shooting up my chest. I couldn’t breathe…I felt like I was having a heart attack.” The on-call nurse at her clinic suggested she take Tylenol for the pain. When the pain failed to subside several hours later, her roommate took MC to the emergency room and called her clinic to let them know. An ultrasound revealed MC’s ovaries were bleeding and her hemoglobin levels were low. When her physician arrived, he advised her that surgery may be required but she was unsure as to why. MC remained in the hospital for 3 days, where she was given Dilaudid, Percocet, and Hydrocodone for ongoing pain. Upon discharge, the doctor informed her that had her condition not stabilized, he would have needed to remove her ovaries.

Of the remaining three listed in Table 2, donors no. 64, 142, and 404 had all undergone repeat donations. For the cycles in which they report experiencing critical OHSS, donor no. 64 was unsure as to which trigger shot she had received, donor no. 142 reported using an hCG trigger, and donor no. 404 reported receiving a combined hCG-Lupron trigger. All reported between 16 and 35 oocytes retrieved for the critical OHSS cycles. Further details on each case are as follows.

Donor 64 reported critical OHSS on her 13th and final donation cycle. Only mild-to-moderate symptoms were reported on most of her earlier cycles, where she reported between 20 and 35 eggs retrieved on each cycle. By cycle 11, she reported menstrual irregularities and ovarian cysts with increasing ovarian pain compared to her earlier cycles. Within 1 week following her 13th cycle, she experienced difficulty breathing, rapid weight gain, and extreme pain in her ovary. She went to the hospital where she underwent paracentesis and removal of an ovary due to ovarian torsion. She remained in the hospital for several days. Within 1 year following her final donation, she was diagnosed with polycystic ovarian syndrome (PCOS).

Donor 142 underwent a total of three donation cycles. Her third cycle was a “shared donation,” in which she was to keep half of the oocytes produced for herself. This donor shared her medical records, which indicated a first cycle resting antral follicle count of greater than 20 follicles in her right ovary, greater than 15 in her left, and “very PCO appearing” ovaries. Her first cycle protocol included Lupron 5, Gonal F 150, and Menopur 75 daily for 11 days followed by hCG trigger; 25 eggs were retrieved. Prior to triggering, her E2 level was reported at 14,387. Within 24 h post-retrieval, she returned to the clinic reporting vomiting, dizziness, nausea, and bloating and received 2 l of saline and 4 mg of Zofran. Her second donation protocol included Lupron 5, rFSH 113 + hMG 75 for 9 days, a pre-trigger E2 level of 7669, with an hCG trigger. Her third shared cycle followed a similar medication protocol but for 11 days, rather than 9, with an hCG trigger and an E2 level of 6879 prior to triggering. Clinic records indicate 20 follicles in the right ovary and 19 in the left prior to retrieval. Of 22 oocytes retrieved, five were frozen for the donor and 17 donated to a recipient. Following her final cycle, she reported a “kidney blood clot” (renal vein thrombosis) and acute renal failure and had been admitted to the ICU. A follow up interview with this donor revealed that hospital nursing staff told her she had also experienced cardiopulmonary arrest and had been resuscitated. She remained in the hospital for an entire week and was prescribed blood thinners for the next 6 months. In a survey text box, she states:

My third and final and most traumatic donation, I told my doctor I thought the trigger med amount they were having me do was too high from my previous experiences, but because I was a few years older and I was also receiving a split donation for myself, I feel like that was more of a concern to the doctor to get more eggs, than [concern] for my health. After that donation I had a blood clot in my kidney, stayed in the hospital for a week and was on blood thinners for 6 months afterwards and went to a kidney doctor for 3 years after to check up on it. Luckily everything is now fine.

Donor 404, mother of 2, underwent her first donation at 26, did two donations per year until age 30, and underwent her final donation a year later at 31, for a total of eight cycles, all at the same clinic. On her second cycle, she reported being hospitalized shortly after administering her final dual trigger injection, but before oocyte retrieval, due to extreme pain, difficulty breathing, and gaining about “15-20 pounds of water weight in less than 24 hours.” She described feeling like “someone was standing on my lungs.” During a follow-up interview, she stated that the ultrasound conducted in the hospital revealed her ovaries were so swollen with follicles they had twisted around behind her uterus and were touching each other. At the hospital, paracentesis was performed, and she was released the following day. One day later, she went through the retrieval procedure when more fluid was drained. Her survey indicated she produced between 12 and 16 follicles on each of her eight cycles, which seemed unusual given the symptoms she described. During the interview she clarified: “They told me that they only made about 12 embryos on each cycle but that I usually had about 16-20 very healthy large egg follicles on each side, so on the survey I only included the embryos they told me about.”

Despite having severe OHSS on cycle 2, she continued to donate because she was going through a divorce and needed the money to support her children. After cycle 3, her blood pressure started measuring higher than usual. With each subsequent cycle, she began to experience other symptoms, such as gradually increasing blood pressure, occasional bouts of aphasia, nosebleeds, minor seizures and shaking, and transient ischemic attacks, but she was not sure if these were connected to her egg donations, and she did not relay this information to the clinic. She planned to stop after her seventh cycle, but almost a year later, the clinic called her for a “sibling cycle,” and she felt like she could not say no. Following her eighth and final cycle, her blood pressure started “spiraling out of control,” measuring at 175/115—stage 3 hypertension. She had fainted at home, was sent by ambulance to the hospital, where she remained for the better part of a week.

### Trigger shot by time since last donation

**Fig. 4:**
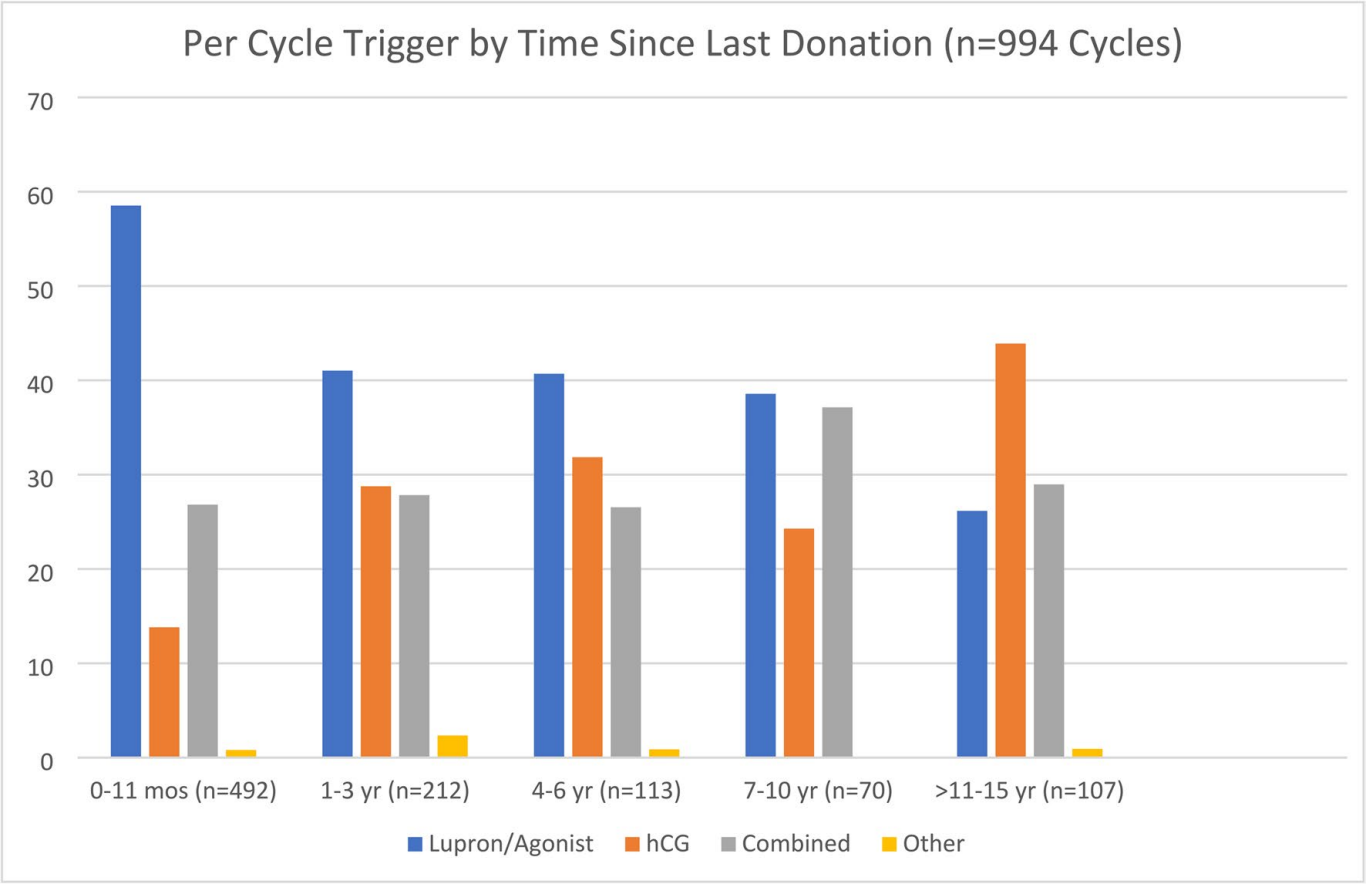
Among donors whose most recent cycle was completed within 11 months prior to taking the survey, 58.5% (*n* = 492) of cycles reported a GnRHa trigger compared to 26.2% (*n* = 107) for donors who completed cycles 11 or more years prior

With the known improved safety of GnRH agonist triggers, it is often assumed that severe OHSS is no longer a concern due to the availability safer protocols. A further analysis of 994 donation cycles in which trigger type was reported, regardless of whether the donor experienced OHSS, demonstrates an increased use of GnRH agonist triggers over time, along with an accompanying decrease hCG-only triggers and dual triggers remaining somewhat constant. As seen in Fig. 4, among donors whose most recent cycle was completed within 11 months prior to taking the survey, 58.5% (*n* = 492) of cycles reported a GnRHa trigger compared to 26.2% (*n* = 107) for donors who completed cycles 11 or more years prior.

## Discussion

Examining safer stimulation protocols for oocyte donors is of primary concern, especially considering donors undergo this process with no medical benefit to themselves. In our data, more than 12% of all donors surveyed reported experiencing severe OHSS, in addition to the 1.38% reporting critical OHSS, in at least one of their donation cycles. While we cannot know for certain if the donor population in this study is a representative sample of the broader population of US compensated egg donors, our findings are consistent with investigations examining OHSS rates in donor and non-donor populations undergoing controlled ovarian stimulation [5, 9–11, 21]. To our knowledge, this is the first study to explore possible linkages between trigger type, oocytes retrieved, and prior OHSS history in oocyte donors.

Some have suggested that the use of milder stimulation protocols for oocyte donors be mandatory [22]. As Bodri et al. demonstrate, “The risks of OHSS can be substantially reduced by specific stimulation protocols, which include GnRH agonist triggering” [23]. Galindo et al. similarly demonstrate in a prospective clinical trial with 257 donors that donor and recipient outcomes are comparable for both hCG and GnRH triggers, and the risk of OHSS for donors is considerably reduced using a GnRH antagonist protocol with GnRH agonist triggering [24]. Our survey findings from donor self-reports are consistent with the findings of both studies; donors who report receiving a GnRH agonist trigger also report much lower rates of severe OHSS.

It is well-established that use of an agonist trigger, over a trigger containing hCG, both reduces risk for OHSS in fertility patients and egg donor populations without jeopardizing fertilization, implantation, or pregnancy rates [13, 25–30]. In their prospective study of 339 IVF patients undergoing COS, Jayaprakasan et al. demonstrate that the OHSS risk for cycles in which fewer than 20 follicles develop is very low (< 0.1%), but if greater than 20 follicles develop, OHSS risk increases to approximately 15% [11]. With a specific focus on oocyte donors, we similarly find that the risks for severe OHSS increase according to the number of eggs retrieved across all trigger types but are still lower for those who receive a GnRH agonist trigger.

Our analysis revealed a clear upward trend in the use of GhRH agonist administration to trigger final oocyte maturation among donors responding to our survey, accounting for a reported 58.5% of donor cycles in 2018 or later versus only 26.2% of donor cycles prior to 2010, as expected. The results among our relatively small sample of donor egg retrievals likely underestimate more widespread trends. One large-scale evaluation of oocyte donor treatment practices reported that by 2015, 95% of oocyte donation cycles used GnRH antagonist protocols for ovarian stimulation, and approximately 70% of all donation cycles were triggered with GnRH agonists (versus only 26% in 2009), but we are aware of no more recent data addressing this issue [31]. However, the apparent continued existence of a substantial proportion of triggers including hCG in relatively recent donor egg retrieval cycles suggests that despite the advantages of GnRHa-only triggers, they still remain underutilized among the donor population who are at particularly high risk for severe OHSS.

Although an agonist trigger is associated with reduced OHSS risk, and live birth outcomes are equivalent, it does not appear that the adoption of this protocol for current or recent donors is as prevalent as would be expected. Our analysis of reported trigger type by time indicates that slightly over half of the women who donated eggs within a year prior to taking the 2019–2020 survey reported receiving a GnRH agonist trigger. This could be one explanation as to why, as Rotshenker-Olshinka et al. address, OHSS hospitalization rates have not declined, as would be expected, if safer stimulation protocols were used more widely [20]. Further investigation is warranted into practitioner protocol preferences and reasons for not adopting antagonist cycles with agonist triggering for the oocyte donor population.

A prior history of severe ovarian hyperstimulation syndrome has long been regarded as a risk factor for additional episodes of severe OHSS. However, the original source of evidence for this supposition is unclear. In the words of a 2000 Modern Trends review published in Fertility & Sterility, “there appears to be general recognition that patients with a history of OHSS are at risk for recurrence in subsequent COH cycles.” However, the authors provided no supporting evidence for this statement. The 2008 ASRM practice committee guidelines on OHSS list “previous episodes of OHSS” as a risk factor, but none of the references cited in support of the risk factor list evaluated this connection. Several other prominent reviews published over the past decade have also claimed a history of OHSS as a risk factor for future OHSS, but none of the supporting refences cited by these reviews specifically evaluated this relationship [30, 32–34]. In the latest draft of ASRM practice committee guidelines on prevention of moderate and severe OHSS, there is no mention of prior OHSS as a risk factor despite systematic review [12]. A history of prior OHSS is also not mentioned as a concern in the 2020 ASRM/SART committee opinion on repetitive oocyte donation, which states only that severe OHSS is expected to occur in no more than 1 to 2% of donation cycles [35].

Drawing on donor self-reports, we provide the first evidence to confirm this long-held belief, noted by numerous reviewers of the subject, that a history of severe OHSS is a significant risk factor for reoccurrence of severe OHSS in subsequent cycles of ovarian stimulation and oocyte retrieval [12, 30, 32–34, 36]. Our results demonstrate that the severity of OHSS symptoms experienced during the first oocyte retrieval cycle is highly predictive of OHSS severity in subsequent cycles by the same donors. Most donors (approximately 85%) who experienced only mild or no OHSS symptoms in their first donation cycles also reported no more than mild OHSS in their second cycles as well. In contrast, 36% of all donors who underwent second cycles after having severe OHSS in first cycles experienced severe OHSS again in second cycles, more than 7 times the frequency of severe OHSS after a first cycle of mild or no OHSS, and more than 4 times the frequency of severe OHSS after a first cycle of moderate OHSS. While changing the medication protocol to one which includes an agonist-only trigger could reduce severe OHSS risk in subsequent donations, our data indicates that this does not appear to be consistently happening. Given such high estimated chances of recurrent severe OHSS, it is worth considering that a single episode of severe OHSS might be a reason to disqualify a donor from future donation cycles.

Case studies of repeat donors who have had at least one episode of severe OHSS provide further insights. In many cases, differences in the severity of OHSS symptoms among cycles by the same donor appear to be explainable by changes in trigger medications or differences in the response to stimulation (i.e., oocyte numbers). However, in many other cases, clinically significant variation in OHSS severity observed within donors could not be explained by differences in trigger medications or oocyte yields. A few donors reacted contrary to expectations, exhibiting more severe symptoms of OHSS when reportedly triggered with less or no hCG, or with retrieval of significantly fewer oocytes. Similarly, in two of the four reported cases of critical OHSS, fewer than 20 oocytes were retrieved. Hence, other unidentified risk factors may have come into play. These cases highlight the significant element of unpredictability of OHSS. Severe OHSS can occur unexpectedly in any patient undergoing ovarian stimulation with gonadotropins, regardless of the trigger medication used, the follicular response to stimulation, or history of OHSS symptoms in previous ovarian stimulation cycles.

At first glance, the four cases of Critical OHSS reported here were somewhat puzzling. While two of the four reported having polycystic ovaries (no. 64, 182), for the cycles on which they reported Critical OHSS, donor no. 404 reported only between 16 and 22 oocytes retrieved—a number that is considered relatively safe. A follow-up interview revealed that this donor had understated the number of oocytes retrieved on her survey—only counting the number of embryos created not eggs retrieved—and that she had actually produced almost double that amount for each cycle. Donor no. 404 did not indicate PCOS in her survey or interview, but we also cannot rule out that possibility. It is also concerning that donor no. 404 continued to undergo repeat donations despite having been hospitalized after her second cycle and considering her other symptoms. Both donor no. 64 and 404 also report having completed between eight and 13 cycles, well above the ASRM recommended number of no more than six. These cases reveal the importance of including qualitative data, rather than relying on survey results alone.

The existence of half a dozen donors who underwent multiple ovarian stimulation cycles with little or no symptoms of OHSS before experiencing severe OHSS in one or more later cycles, unexplainable by either trigger type or follicular response, suggests an intriguing possibility. It is conceivable that undergoing multiple cycles of ovarian hyperstimulation primes the system in such a way as to increase OHSS severity in later cycles, regardless of protocol or ovarian response. At the extreme, one donor (no. 64) reported experiencing no OHSS symptoms during her first ten donations (eight of which were hCG triggered), followed by moderate, mild, and critical OHSS in her next three donations. And donor no. 404 reported other symptoms increasing in severity—such as bloating, high blood pressure, and transient ischemic attacks—with each subsequent cycle. To evaluate this possibility would require a much larger sample of oocyte donors undergoing multiple repeat donations, inclusion of clinical records with associated OHSS diagnosis and treatment, and other cycle characteristics of each donation cycle. This is beyond the scope of the current study.

## Conclusion

This study examines donor self-reports of OHSS severity and corresponding trigger medication, oocyte cohort size, and history of OHSS symptoms in previous stimulation cycles. Despite the limitations of this study, this is the most comprehensive investigation we know of on OHSS in oocyte donors. The research has significant implications for clinical practice. Data presented here can help inform clinics on how to improve donor care and reduce risk for complications, thereby also improving donor satisfaction and safety. Common counseling practices informing donors that the “risks are less than 1%” only appear supported by our data in cases where donors receive a GnRHa trigger and report fewer than 30 oocytes retrieved per cycle or in hCG triggered cycles where fewer than 10 oocytes were reported. Study findings also indicate that donors who had prior experience with severe OHSS, or who have PCOS, may not be good candidates for repeat donations. It is also significant that many donors, including those who experienced severe to critical OHSS, underwent well-beyond the ASRM recommendation to undergo no more than 6 cycles. It may be useful to consider ways to prevent donors from undergoing cycles beyond the ASRM guidelines. This investigation can help reduce important gaps in knowledge regarding information specific to egg donors, who may be at increased risk due to younger age, often lower BMI, and are selected in part because of their high resting antral follicle counts and ovarian response to stimulation medications.

The strengths of this study include the range of sources from which donor-participants were recruited, the breadth of survey questions, the ability to cross-check donor responses for reliability in their assessment of OHSS experiences, and the large number of participants with a range of experiences and time since last donation. Single-clinic studies are limited in their generalizability of data as different clinics use different stimulation protocols and procedures. By recruiting donors from a range of sources—including numerous clinics, egg donation agencies, egg banks, online egg donation groups, and word of mouth—we were able to capture a wider range of donor experiences. This is the largest study we know of to date where egg donor participants were not all recruited from a single clinic or source.

We acknowledge some limitations of this study. The retrospective self-report survey design could potentially introduce perception or recall bias. However, half of all respondents donated within a year, and two-thirds of all donors completed the survey within 3 years of their last donation cycle, and thus, their experiences were relatively recent. The few more delayed surveys did not exhibit any divergent characteristics that might have substantially altered results or conclusions. We also focused our analysis on the distinction between severe (or worse) OHSS characterized by prolonged severe physical distress and the need for medical interventions including hospitalization and abdominal fluid drainage versus moderate or milder OHSS which, while uncomfortable, resolves spontaneously without intervention in less than a week. We believe this is a distinction that donors are likely to recall with relatively high accuracy.

Donors who underwent multiple cycles may also not accurately remember which trigger type was associated with which cycle, which could account for some of the unexpected findings. Since respondents self-selected their participation in the survey, they may not have been representative of oocyte donors in general. It is possible that donors who experienced worse OHSS symptoms may have been more likely to respond to the survey and also may have been more likely to recall adverse events than those who experienced less severe/no symptoms. To enhance accuracy, prospective research is clearly needed following donors throughout the donation process, including pre- and post-donation surveys of donors’ experiences and more comprehensive post-donation clinical follow-up of potential OHSS symptoms. Nonetheless, reports of OHSS in our survey population are consistent with findings from other studies, providing some confidence in data reliability.

This is the only study we know of to date to examine donor-reported experiences of OHSS by trigger type and number of oocytes produced per donation cycle. It is also the only study we know of that evaluates the degree to which prior OHSS experience may be associated with future risk for this condition, among oocyte donors or any other population undergoing repeated cycles of controlled ovarian stimulation of oocyte retrieval and in vitro fertilization. This research is significant because OHSS is a preventable, potentially life-threatening iatrogenic condition that can be largely avoided if appropriate measures are taken to minimize risk. Our findings illuminate how trigger type, quantity of oocytes produced per cycle, and prior OHSS experience all contribute to heightened risk for severe or even critical OHSS. In the interests of donor comfort and safety, these factors should be considered when determining COS protocols and donor suitability for subsequent cycles.
